# Chemical Interactions Involved in Insulin Receptor Activation: Scoping Review

**DOI:** 10.2174/0115733998373981250930135544

**Published:** 2025-11-03

**Authors:** Sofia Fernandes Coriolano Araujo, Stuart Handerson Rodrigues Costa, Antonio Souza Araujo

**Affiliations:** 1 Faculty of Medical Science, University of Pernambuco, Recife, 50100-130, Brazil;; 2 School of Chemistry and Molecular Bioscience, The University of Queensland, Brisbane, Australia;; 3 Institute of Chemistry, Federal University of Rio Grande do Norte, Natal, Brazil

**Keywords:** Insulin receptor, phosphorylation, tyrosine, kinase, insulin signaling, metabolic disease

## Abstract

This work overviews some complex molecular interactions and phosphorylation events in the insulin receptor (IR) signaling pathway and explains its central role in metabolic control, which starts from the synthesis and secretion of insulin by pancreatic β-cells under elevated blood glucose. The triggered pathway coordinates a cascade of molecular processes that results in the activation of primary metabolic functions. Insulin bound to its receptor starts a sequence of events, such as the autophosphorylation of the receptor β subunit for initiating downstream signaling cascades, glycogen synthesis, and the appropriate regulation of lipid metabolism. The complexity and specificity of the signaling pathway involve insulin receptor substrates, phosphatidylinositol-3-kinase (PI3K), and protein kinase B (PKB). A detailed molecular interaction analysis in the IR has pointed to the crucial role of some residues and structural elements necessary for enzymatic functionalities and substrate binding. These factors include the kinase domain of the IR with specific amino acid residues, and subsequent activation of downstream signaling proteins. The structural changes on phosphorylation promote the binding of SH2 domain-containing adaptor proteins, which lead to the initiation of multifunctional signaling complexes central to insulin signal transduction. These molecular mechanisms provide insight into pathophysiology relating to metabolic diseases and potential treatment targets. This review aims an understanding of insulin receptor operation, elucidating the molecular intricacies behind the eventful metabolic insulin signaling pathways and highlighting possible research and therapeutic development in the field of medicine.

## 1 INTRODUCTION

Glucose and amino acids have been viewed as substrates for the generation of molecules and precursors for the biosynthesis of macromolecules. These nutrients also function as signaling molecules in functionally diverse signal transduction pathways. Insulin is a hormone that regulates blood glucose in the body. The control of glucose levels and insulin resistance remains a significant challenge in the effective treatment of diabetes. There are only a few reviews exploring the chemical interactions involved in insulin receptor (IR) activation and its role in glucose control. Thus, based on a systematic approach and considering literature, this review aims to comprehensively describe some chemical molecular processes, involving the insulin receptor, contributing to the development of therapeutic approaches to treat these health conditions. Therefore, this review is expected to significantly contribute to the advancement of scientific biomedical knowledge and improvement of metabolic health care.

Recently, molecular interactions concerning the direction of glucose levels in blood plasma constitute one of the elemental pillars of human metabolic homeostasis and have been receiving more attention, becoming attractive for research in many disciplines [1-3], such as biotechnology, organic biochemistry, and medicine. This balance is kept up through a complex arrangement of atomic signals, with insulin standing out as the primary operator [4]. This peptide hormone is synthesized by pancreatic β-cells in response to an increment in blood glucose levels [5], playing a fundamental part in controlling carbohydrate, protein, and lipid metabolism within the body. The atomic structure of insulin is shown in Fig. (**1**) [6].

For a better understanding of the molecular mechanisms involved in the beginning of the insulin signaling pathway, it is crucial to go back to the production and release of the hormone as well as its effects on specific tissues. The pathway of glucose and insulin in the human body demonstrates an intricate connection between various organs and systems, resulting in well-coordinated and effective interactions leading to changes in blood sugar levels [7].

After entering the blood, insulin binds to its designated receptors on target cells, setting off a series of chemical reactions within the cell that regulate vital metabolic functions [8]. These tightly controlled molecular events in the insulin signaling pathway oversee essential metabolic processes. They begin with the interaction of insulin with its membrane receptor, a glycoprotein composed of two α subunits and two β subunits. This interaction causes the autophosphorylation of the β subunit of the receptor, activating its intrinsic tyrosine kinase activity [9].

The activation of the insulin receptor subunits stands out as the main action that starts phosphorylation events and subsequent activation of some of the key intracellular proteins [10, 11]: insulin receptor substrates (IRS1-4), phosphatidylinositol-3-kinase (PI3K), and protein kinase B (AKT/PKB). The phosphorylated IRS proteins interact with the SH2 domain of the regulatory subunit of PI3K, activating this enzyme [12]. Then, PI3K catalyzes the conversion of phosphatidylinositol 4,5-bisphosphate (PIP2) to phosphatidylinositol 3,4,5-trisphosphate (PIP3), a second messenger for AKT/PKB activation [13, 14]. AKT/PKB is essential in controlling different metabolic functions, for example, moving glucose transporter 4 (GLUT4), producing glycogen, storing fat, and blocking gluconeogenesis [15].

Moreover, insulin signaling is essential in regulating cell growth and differentiation, ultimately impacting tissue growth and function [16-20]. This pathway started when IRS binds to growth factor receptor-bound protein 2 (GRB2). It causes the activation of the son of seven less protein (SOS protein), which activates the rat sarcoma virus protein (RAS), leading to a series of other phosphorylation events that activate the serum response factor (SRF) and ETS like-1 (ELK-1) transcription factors for gene expression and cell growth [21-23]. The Fig. (**2**), represents a brief scheme of the insulin signaling pathway [24].

The comprehension of these complex molecular processes, mainly involving the insulin receptor, opens diverse perspectives on the pathophysiology of metabolic disorders and has the potential to contribute to the development of more precise and effective therapeutic approaches to treat these health conditions. This paper aims to investigate in detail the complex molecular interactions and specific phosphorylation that occur in the insulin receptor, thereby providing a deeper understanding of the metabolic mechanisms regulated by the hormone in analysis.

## 2 MATERIALS AND METHODS

### 2.1 Study Design

For the study design, a detailed description of the observational study and the methodology used is provided. Thus, this review adopted an observational, scoping design aimed at mapping and synthesizing the existing literature on the chemical interactions involved in insulin receptor activation. The study does not involve the collection of primary experimental data; instead, it systematically compiles and evaluates findings from previously published research. Guided by the PRISMA flow diagram, this approach ensures a transparent and reproducible process of literature identification, selection, and appraisal, thereby facilitating a comprehensive understanding of the molecular mechanisms underlying insulin signaling.

The methodological procedure used in this review included systematic research in the literature, based on three research databases: PubMed/Medline, Scopus, and Web of Science/Clarivate. The searches were carried out for articles available in the literature, with emphasis on the period from 2019 to 2024. During the search, the keywords contained in the article were used, and then the word “chemical interactions” was added. Thus, the combinations were as follows: “chemical interactions” AND “insulin receptor” AND “phosphorylation” AND “tyrosine” AND “kinase” AND “insulin signaling”. Such combinations and variations were used to expand the possibilities of finding published articles related to the topic.

To search for publications, articles were selected in English from the aforementioned research databases. During the review, studies on chemical interactions in insulin receptor activation - available as full text and accessible through the networks of the Federal University of Rio Grande do Norte and the University of Pernambuco *via* the CAPES platform (https://www.periodicos.capes.gov.br/) - were considered eligible. The articles were selected and downloaded to verify their relationship with the topic of the study. Initially, the articles were screened by title and abstract, and then read in full to define their inclusion. At the end of the searches using the article’s keywords, a total of 794 articles were found (455 in PubMed/Medline, 240 in Scopus, and 99 in Web of Science/Clarivate). After considering the relevance of chemical interactions in the context of insulin receptor activation, the number was reduced to 30 articles (19 from Scopus and 11 from PubMed/Medline). Following the selection process and application of the eligibility criteria, these articles were included in this scoping review, as detailed in the PRISMA diagram (Fig. **3**).

## 3 RESULTS

### 3.1 Structure and Main Function of Insulin

Insulin relies on the regulation of blood glucose levels and the energy metabolism of the human body. Made up of a polar, charged, and large polypeptide molecular structure consisting of 51 amino acids, insulin is unable to independently cross the lipophilic, apolar cell membrane of insulin-dependent tissues, requiring a receptor. The polypeptide insulin structure is displayed in Fig. (**4**).

The primary function of the anabolic hormone is to increase and speed up the uptake of glucose by cells, particularly in insulin-dependent tissues: adipose tissue and striated muscle tissue (skeletal and cardiac). The anabolic role of insulin is demonstrated by its effects on adipose tissue, where it promotes fat synthesis and storage, and muscles, where it increases glucose uptake and supplies energy during muscular activity [25]. For the body's systems to function correctly and to maintain glycemic balance, these effects are essential. Some metabolism problems, for example, diabetes mellitus, might result from low or inadequate quantities of this peptide.

### 3.2 Synthesis and Secretion of Insulin

Insulin synthesis begins in the nucleus of beta cells, where the genetic code of the petite arm - p arm (short arm) of chromosome 11 is transcribed into messenger RNA [26]. This RNA is then translated into amino acids to form pre-proinsulin, which is then processed to become proinsulin and, subsequently, active insulin, along with peptide C. The insulin hexamer, widely used in storage, is formed by six insulin molecules associated with a zinc ion present in the surrounding environment. Finally, in the secretory vesicle, peptide C is secreted along with insulin in equimolar amounts. Additionally, it is important to emphasize that insulin participates in both endocrine and paracrine regulation [27]. Paracrine regulation occurs when a cell releases chemical signals to nearby cells of the same organ or tissue, allowing for more precise cellular communication and better regulation of metabolic processes.

The vital function of insulin in the metabolic and energy regulation of the human body is demonstrated by these complex and interconnected mechanisms [28]. Next, the essential molecular interactions for the activation of the insulin receptor in insulin-dependent tissues will be discussed.

### 3.3 The Insulin Receptor (IR)

The heterotetrameric insulin receptor (IR) stands as a complex protein assembly, comprising two unique pairs of units, totaling four, including two extracellular alpha (α) units and two transmembrane beta (β) units, all of them containing domains that exert specific and coordinated effects that mediate insulin signaling [29]. There are disulfide bridges within each dimer, cross-linked linking the α and β subunits. In addition, there is an extra disulfide bridge that connects the two α subunits of adjacent dimers, so the tetrameric structure joined forms the complete and functional structure of the receptor.

The β units of the IR showcase an extracellular segment, a transmembrane part, and an intracellular domain armed with kinase activity [30]. The extracellular segment engages in binding with the α units and insulin, while the transmembrane region, through a conformational shift towards the alpha helix, secures the receptor onto the cell membrane. This structure reflects a notable secondary protein framework, typified by a helical formation where the polypeptide chain coils around an imaginary axis. These hydrophobic structures contain residues with nonpolar side chains, such as alanine, leucine, isoleucine, and valine, well-suited for the lipid-rich vibe of the cell lipid bilayer membrane [31]. Their chains are predominantly carbon and hydrogen atoms, lacking polar functional groups, preventing hydrogen bonding or strong water interactions. The intracellular domain includes the kinase domain, which, once activated by insulin binding, initiates signaling cascades regulating cellular metabolism [32].

The insulin receptor contains fibronectin type III (FnIII) domains in both subunits [33]. There are two of these, named F1 and F2. An insert of considerable size is present in the F1 domain, spanning the α/β junction, and the F2 domain is mainly present in the β subunit. They range in length from 90-100 amino acid residues and contain a distinctive array of antiparallel β-sheets that give rise to what is known as a β-sandwich. This represents a highly stable structure involved in mediating protein-protein interaction. The F1 domain, along with its large insert, has a role in the conformation and assembly of the insulin receptor. This insert influences the conformation and flexibility of the receptor, which further elicits an effect on insulin binding, eventually resulting in receptor activation. The F2 domain present in the β subunit helps in structural stability and signal transduction that occurs during insulin binding.

On the other hand, the alpha subunits consist of two extracellular domains linked by a disulfide bridge. The bridge forms between sulfur (S) atoms of cysteine (Cys) residues within each extracellular domain [34]. Cysteine contains a thiol group (-SH), wherein oxidation of adjacent cysteine residues forms a covalent disulfide bridge (S-S bond) [35]. These bridges stabilize the structure of the alpha subunit, and they are represented by the following equation:

Cys-SH + Cys-SH → Cys-S-S-Cys + 2H^+^ + 2e^−^       (01)

Additionally, there are two lobes that are ligand-binding in α subunit domains: L1 and L2. These are a pair of homologous duplicated domains and are located in the extracellular section of the insulin receptor. Both domains have four or five copies of one repeated structural motif-helix-turn-strand [36]. They are made up mainly of amino acid residues, which form the α-helices and β-sheets. They are required, in association with beta turns, to retain a secondary structure. The precise make-up cannot be determined, but a common entity is a mix of hydrophobic and hydrophilic amino acids that attain stability and activity in the domain.

The L1 and L2 domains are the most critical in determining insulin binding because they contain the primary insulin-binding sites, which enable the hormone molecule to anchor tightly to the receptor. In this context, it is through the cysteine-rich domain that interaction between the L1 and L2 domains is made in the formulation of an overall extracellular part of the receptor and allows the effective binding of insulin. Three repetitions of a specific structural motif characterize this domain, and it possesses a high density of Cys residues, forming many intramolecular disulfide bridges, which are responsible for the stability and rigidity of the structure. The disulfide bridges in the cysteine-rich domain are essential resources to bring about the three-dimensional shape necessary for interaction with insulin and other proteins [37]. These covalent bonds between cysteine residues are formed during protein synthesis and folding, facilitated by specific enzymes such as protein disulfide isomerases.

The amino acid residues constituting the ligand-binding lobes of the alpha subunit harbor distinct chemical traits, facilitating complementary interactions with specific insulin polypeptide residues to stabilize its tridimensional structure or bonds [38]. These interactions encompass hydrogen bonding, hydrophobic interaction, and electrostatic interaction.

### 3.4 Hydrogen Bonding

Hydrogen bonding is chiefly established between hydrogen and electronegative atoms (notably oxygen, nitrogen, or fluorine), resulting in a notable electronegativity discrepancy during hydrogen interaction, eliciting pronounced attraction [39]. When considering interactions between the insulin receptor alpha subunit's ligand-binding lobes and insulin's polypeptide structure, hydrogen bonding typically occurs between hydrogen and oxygen or nitrogen functional groups of amino acid residues capable of donating or accepting hydrogen protons [40]. For example, within the ligand-binding lobes, -NH and -OH groups of amino acids such as asparagine (Asn), serine (Ser), threonine (Thr), lysine (Lys), and glutamine (Gln) facilitate hydrogen bonding with the carboxyl (COOH) and amino (NH_2_) groups of amino acids present in insulin polypeptide chain, such as aspartic acid (Asp), glutamic acid (Glu), lysine (Lys), and arginine (Arg).

### 3.5 Hydrophobic Interactions

In contrast, hydrophobic interactions occur between nonpolar regions of molecules, where hydrogen and carbon atoms are the main participants, as they jointly exhibit low electronegativity differences and, consequently, lesser attraction [41]. In the ligand-binding lobes, amino acid residues participating in hydrophobic interactions, such as alanine (Ala), valine (Val), leucine (Leu), isoleucine (Ile), phenylalanine (Phe), methionine (Met), and tryptophan (Trp), typically possess hydrophobic side chains, akin to insulin [42].

These residues contribute to insulin's hydrophobic regions due to their shared amino acid constituents. As hydrophobic side chains of ligand-binding lobe amino acid residues approach insulin's surface lipophilic regions, hydrophobic interaction ensues [43]. This interaction type is particularly pertinent in aqueous environments, encompassing the cytosol inside the cell and the plasma outside of it. In the examined scenario, both ligand-binding lobes and insulin's lipophilic regions assemble, forming aggregates or more condensed structures [44]. This phenomenon arises from water molecules encasing these nonpolar regions, presenting a thermodynamically unfavorable situation. Hence, hydrophobic molecules tend to aggregate, minimizing water contact and reducing the formation of energetically costly “cages.”

### 3.6 Electrostatic Interactions

Within the insulin receptor alpha subunit's ligand-binding lobes, amino acid residues with opposing charges establish electrostatic interactions with corresponding residues in insulin's polypeptide structure [45]. Such interactions involve specific atoms of amino acid functional groups. For example, lysine (Lys) and arginine (Arg) feature positively charged amino (NH_2_) groups, electrostatically interacting with negatively charged groups, such as carboxyl (COO-) groups of aspartic acid (Asp) and glutamic acid (Glu) residues. Both insulin and ligand-binding lobes encompass chains with positive and negative groups, facilitating mutual electrostatic interaction between opposite charges of their peptide chains. These bonds predominantly form between nitrogen atoms of amino groups and oxygen atoms of carboxyl groups [46].

### 3.7 Conformational Dynamics on the Insulin Receptor

As insulin molecules bind to the alpha subunit of the IR through diverse interactions and mechanisms, a conformational change occurs in the receptor's beta subunits, which acts as a switch activating enzymatic activity [47]. This activation leads to the approximation of the tyrosine kinase domains present in the beta subunits, forming a catalytically active configuration of the tyrosine kinase enzyme. This proximity is essential for facilitating the efficient transfer of phosphate groups to tyrosine residues on substrate proteins.

At first, prior to insulin attachment, the ectodomain of the IR takes on a U-shaped structure, which shows the receptor not active yet, with the alpha subunits positioned to keep the beta subunits separated. When insulin attaches to it, the receptor's ectodomain changes its shape significantly, going from a U-shape to a T-shape [48]. This adjustment, shown on (Fig. **5**), decreases the gap between the beta subunits, bringing them nearer and allowing their self-phosphorylation and activation of the initial tyrosine kinase, thus triggering internal signaling.

This structural alteration of the IR also brings about a restructuring of the helices that lie in the transmembrane regions. This region is described as any part of a protein that goes across the cell membrane lipid bilayers and connects the ectodomain and the endodomain [49]. The amino acids can rotate, tilt, and twist around the peptide bonds that join them. This allows all the amino acids to move in relation to each other, taking on different 3D shapes of the helix, thereby influencing how neighboring amino acids interact in the sequence of the helix, which leads to changes in its tridimensional configurations.

Direct interactions between amino acids in these helices and the lipids in the lipid bilayer may, therefore, facilitate specific movements of these helices [50]. Hydrophobic amino acids tend to be located at the center of a helix. They are, thus, likely to interact with the membrane lipids, whereas the polar amino acids might interact with the aqueous environment.

## 4 DISCUSSION

### 4.1 Activation of the Insulin Receptor

Upon binding to either of the two alpha subunits of a surface insulin receptor, a complex series of molecular structural changes are set in motion within this receptor [51]. These structural rearrangements set off a chain reaction of molecular events that culminate in the activation of the enzymatic activity of the receptor.

This process, once proximity-induced dimerization of β-subunits has occurred, enables trans-autophosphorylation to occur and is a critical primary event toward full activation of the receptor [52]. For example, the same autophosphorylation site within the beta subunit kinase domain of Tyrosine Kinase Enzymes includes several tyrosine residues at positions Tyr1158, Tyr1162, and Tyr1163. Tyrosine phosphorylation causes a conformational change in the enzyme receptor, which augments the affinity of the kinase domain for Adenosine Triphosphate (ATP) and protein substrates [53]. It thus becomes necessary to activate catalytic receptors.

### 4.2 Tyrosine Kinase Enzymes

Tyrosine kinase enzymes (TKs) are enzymes responsible for cell signaling, which catalyze the transfer of phosphate groups from ATP to the specific tyrosine residue within proteins. This phosphorylation changes the functional activity of the target proteins, altering their molecular interactions and cellular functions. It leads to the activation or inactivation of enzymes and establishes a multiprotein complex transmitting a signal from the membrane to the cell nucleus [54, 55]. One of these enzymes, a receptor tyrosine kinase (RTK), the insulin receptor, has an intracellular cytoplasmic kinase domain. RTKs are a subfamily of tyrosine kinases that, on activation by extracellular ligands such as hormones or growth factors, dimerize and auto-phosphorylate specific tyrosine residues, enabling the binding to proteins containing SH2 domains, thereby initiating different intracellular signaling pathways [56].

### 4.3 Structure of the Kinase Domain

The kinase domain of the insulin receptor is a highly specialized region of protein with approximately 300 amino acid residues folded to form a complex three-dimensional structure. The section is packed with several α-helices and β-sheets that make up the catalytic core of the enzyme [57]. The α-helices are helical structures that are responsible for the stabilization of the protein through hydrogen bonds between atoms of the polypeptide backbone, which form a rigid structural base required for catalytic function [58]. The spiraling mainly contributes toward maintaining the correct three-dimensional conformation of the domain in such a way that helps in positioning the catalytic and substrate-binding regions appropriately.

The β-sheets are, in contrast with the α-helix, formed by polypeptide chains lying side by side and connected through hydrogen bonds between O-atoms of peptide bonds [59]. This achieves a good benefit from the aqueous core (since the presence of water facilitates these hydrogen bonds), which is crucial for maintaining the overall strength of the protein core. Such structural stability is essential for functional enzymatic activity so that this kinase domain maintains a precise configuration needed for efficient catalytic activity. Together, these secondary structures help the kinase domain of the insulin receptor in the specific and efficient execution of its catalytic functions [60]. The structure of this domain is well conserved for RTKs, pointing to its value during evolution and in various long-aged fundamental cellular processes.

### 4.4 Catalytic Core and Binding Sites

The kinase domain region where phosphorylation reactions occur is called the catalytic core, which possesses two major binding sites: the ATP binding site and the substrate binding site, both forming a deep cleft common to all kinases and to all other receptors for this molecule. This crevice binds ATP and transfers its γ-phosphate by the proper alignment and position of the structure. A series of non-covalent interactions, such as hydrogen bonds and electrostatic interactions, help stabilize the binding of ATP and ensure that proper ATP orientation for the phosphorylation reaction is established [61].

Involved in ATP binding are several amino acid residues, which include Lys1030 and Asp1132, that interact directly with the phosphate groups of ATP and stabilize the molecule, thus facilitating the phosphate transfer. Also, the architecture of the substrate binding site is equally crucial because the tyrosine residues of the substrate are made to orient properly for the phosphoryl group transfer to tyrosine [62]. The accuracy in setting and maintaining these binding sites is critical to the catalytic action of the kinase domain in that tyrosine residue, because phosphorylation will take place efficiently and in a particular manner. The high-affinity binding of ATP to the kinase domain of the insulin receptor and the correct orientation of ATP and substrate in the catalytic core are crucial for the effectiveness of the phosphorylation reaction [63].

### 4.5 Mechanism of Phosphorylation

The phosphorylation of specific tyrosine residues within the kinase domain embodies a perfectly orchestrated series of events [64]. Initially, ATP slots into its designated binding site within the kinase domain. A network of non-covalent interactions—such as hydrogen bonds and electrostatic forces—stabilizes ATP in the proper conformation to transfer its phosphoryl group. Subsequently, the kinase enzyme hydrolyzes the phosphodiester bond connecting the phosphate groups in ATP, releasing an inorganic phosphate (Pi) and converting ATP into Adenosine Diphosphate (ADP). This reaction releases a significant amount of energy, which is then used to form a covalent bond between the liberated phosphate group and a tyrosine residue on the substrate [65].

The γ-phosphate (terminal phosphate) of ATP is transferred to the oxygen of the tyrosine hydroxyl group, forming phosphotyrosine [66]. This process is enabled by the high reactivity of the tyrosine hydroxyl group and the precise spatial configuration provided by the surrounding α-helices and β-sheets. In this reaction, Asp1132 acts as the catalytic base by directly interacting with the tyrosine’s hydroxyl group, while Lys1030 stabilizes the ATP molecule through its amino-group-containing side chain, which interacts with the phosphate groups [67]. The efficiency of phosphate transfer depends critically on the proper positioning of the tyrosine residue within the active site [68]. This intricate interplay illustrates how the three-dimensional structure of the kinase leverages multiple dynamic interactions to achieve an efficient phosphate transfer.

Besides these events, the Mg^2+^ ion is essential for phosphorylation because it holds ATP at the binding site and facilitates phosphate transfer [69]. Mg^2+^ makes direct contact with ATP’s phosphate groups and key catalytic residues, coordinating the beta and gamma phosphates to stabilize ATP and reduce the repulsion between their negative charges. This coordination is crucial for the efficient hydrolysis of the phosphodiester bond and the subsequent transfer of the phosphate to the tyrosine residue. Moreover, Mg^2+^ stabilizes the transition state during catalysis by neutralizing developing negative charges, thereby lowering the activation energy for the reaction. The effectiveness of Mg^2+^ can be attributed to its chemical properties: as a divalent cation with a high charge density and a small ionic radius, it fits snugly into active sites and interacts closely with ATP and catalytic residues. Coordinated in an octahedral geometry (bound to six ligands, such as water oxygen atoms, ATP phosphates, and amino acid side chains), this arrangement maximizes interaction points and stabilizes intermediate states during the phosphate transfer [70].

### 4.6 Molecular Interactions and Key Residues in Phosphorylation

The efficiency and specificity of the phosphorylation of tyrosine residues in the insulin receptor depend on precise interactions between these and various other amino acid residues in the kinase domain, each playing an essential role in the receptor's enzymatic function [71]. A proposed scheme for phosphorylation cascade showing a series of proteins in a pathway is shown in Fig. (**6**).

A critical regulatory element is the steric barrier formed by Phe1151 and Met1153, which prevents ATP from accessing the catalytic site in the inactive (apo) form of the kinase [72]. Phe1151, a phenylalanine residue, contains a large, planar benzene ring that not only engages in hydrophobic interactions with neighboring non-polar side chains but also participates in π-π stacking with nearby aromatic residues. In this stacking interaction, the overlapping electron clouds of adjacent aromatic rings create a stabilizing effect that minimizes the exposure of hydrophobic surfaces to the aqueous environment, thereby reinforcing the inactive conformation [73]. Met1153, a methionine residue, features a thiomethyl group (α-CH_3_ attached to a sulfur atom) whose larger atomic radius imposes additional spatial hindrance. Moreover, the presence of sulfur allows for both hydrophobic interactions and weak van der Waals forces with adjacent residues, further enhancing the steric blockade with Phe1151. Together, these residues create a robust physical barrier that ensures the kinase remains inactive until insulin binding triggers the conformational changes needed for activation.

The necessary structural flexibility of the kinase domain is largely provided by the glycine residues Gly1005 and Gly1152 [74]. Glycine, being the smallest amino acid with a single hydrogen as its side chain, allows for extensive rotational freedom around its peptide bonds without causing steric strain. Gly1005 is located in a region characterized by high mobility, enabling the kinase domain to adopt the various conformations required during the phosphorylation process. Similarly, Gly1152, which resides in the activation loop, a particularly dynamic segment of the protein, ensures that this loop can undergo the critical conformational adjustments required for enzyme activation, facilitating proper substrate and cofactor accommodation [75-77].

Stabilization of ATP binding and the effective transfer of phosphate groups are achieved through the coordinated roles of Lys1030 and Asp1132. Lys1030, found in the N-lobe of the kinase domain, stabilizes ATP by interacting with its phosphate groups *via* strong electrostatic forces and hydrogen bonds. Its positively charged side chain is essential for maintaining the proper orientation of ATP, thereby forming a productive enzyme-substrate complex [78]. In contrast, Asp1132 serves as the catalytic base, directly interacting with the hydroxyl group of the substrate tyrosine. Its carboxylate side chain not only promotes the transfer of the phosphate group from ATP but also forms hydrogen bonds that help orient the tyrosine in the optimal geometry for catalysis [79].

Another crucial feature is the P+1 loop, which plays a significant role in positioning the substrate tyrosine (Tyr1162) for phosphorylation [80]. The P+1 loop, situated immediately adjacent to the phosphorylated tyrosine, is essential for substrate recognition and stabilization of the active site. Trp1175, with its bulky aromatic side chain, anchors the loop through hydrophobic interactions and aromatic stacking; its indole ring fits snugly between residues within the loop, maintaining a conformation conducive to substrate binding [81]. In contrast, Pro1172, owing to its cyclic structure that loops back to bond with its own amino group, restricts the flexibility of the P+1 loop. This rigidity-resulting from proline’s five-membered ring structure-is vital to preserve an invariant and stable loop shape, ensuring that Tyr1162 remains precisely positioned and oriented to receive the phosphate group during catalysis [82].

Finally, Arg1136, located near Tyr1162, plays a vital role in stabilizing the phosphorylated state of the kinase domain. After phosphorylation, the positively charged guanidinium group of Arg1136 forms strong electrostatic interactions, ionic bonds, and hydrogen bonds with the newly attached phosphate group on Tyr1162. This interaction is crucial for maintaining the active conformation of the kinase and ensuring efficient downstream signaling [83].

### 4.7 Conformational Changes and Adaptor Protein Binding

Upon phosphorylation of the tyrosine residues, further conformational changes are induced within the kinase domain, increasing its catalytic activity [84]. These changes are phosphorylation-induced and thus serve as one level of signal transduction within the cell. The phosphorylation sites provide recognition sites for adaptor proteins that contain SH2 (Src homology 2) domains.

SH2 domains have a crucial role in many cellular signaling networks and are commonly found in adaptor proteins. They are specifically known to associate selectively with phosphorylated tyrosine residues on target proteins through a well-defined tertiary structure made up generally of 100 amino acids arranged in a globular fold. Stabilization, dominantly, is done by the internal central β-sheet in this tertiary structure, sandwiched between α-helices, which gives rigidity and stability to the domain. The phosphotyrosine-binding pockets of SH2 domains are conserved and essential in conferring specificity in binding and recognizing tyrosine residues when phosphorylated [85]. Within these pockets, the primary amino acid residues that establish an interaction with the phosphate group of the phosphorylated tyrosyl residue are arginine and lysine. The positively charged side chains of residues in arginine and lysine complement the negatively charged phosphate moiety, facilitating electrostatic interactions that contribute to the specificity and affinity of SH2 domain binding to phosphotyrosines.

This becomes quite important for forming different signaling complexes, which are very dynamic in nature since SH2-mediated recruitment of signaling proteins allows the interaction with other signaling pathways. It is this crosstalk among different signaling molecules that amplifies the insulin signal and produces multiple cellular responses.

### 4.8 Stabilization and Phosphorylation of Specific Tyrosines

Phosphorylation of Tyr1158, Tyr1162, and Tyr1163 is crucial for full insulin receptor activation. Tyrosine is an aromatic amino acid characterized by its phenolic hydroxyl group, critical for phosphorylation [86]. The hydroxyl group can donate a proton, enabling the addition of a phosphate group by kinases. This phosphorylation introduces a bulky, negatively charged phosphate moiety, which significantly alters the electrostatic and spatial properties of the receptor's kinase domain.

In the basal state, the insulin receptor activation loop, where these tyrosines reside, is positioned in such a way that it blocks access to the kinase catalytic site, preventing substrate binding and catalysis. The receptor exists as a dimer, but the activation loop hinders its activity. Upon insulin binding to the extracellular alpha subunits, a conformational change is transmitted through the receptor, bringing the intracellular beta subunits into closer proximity and enabling trans-autophosphorylation.

The initial autophosphorylation occurs at Tyr1158. This is critical because it triggers a conformational change that partially displaces the activation loop, relieving the steric hindrance that obstructs the kinase active site [87]. The structural rearrangement is driven by the formation of new hydrogen bonds and electrostatic interactions between the phosphate group of Tyr1158 and nearby amino acid residues, which stabilize this intermediate conformation.

Tyr1162, upon subsequent phosphorylation, further destabilizes the inhibitory conformation of the activation loop. Phosphorylation at Tyr1162 likely disrupts existing hydrogen bonds and introduces additional negative charges that repel neighboring negatively charged residues, enhancing the displacement of the loop. The final phosphorylation event at Tyr1163 solidifies the extended, active conformation of the activation loop. This extension is stabilized by intramolecular interactions such as hydrogen bonds between the phosphate groups of the phosphorylated tyrosine and between groups from the peptide backbone or side chains of other residues within the kinase domain [88, 89]. Additionally, π-stacking interactions among the aromatic rings of the tyrosine residues and adjacent aromatic residues can further stabilize the active conformation by reducing entropy and optimizing spatial arrangements. The sequential nature of these phosphorylation events is crucial because each step progressively opens the kinase domain, ensuring precise control over the activation process.

These phosphorylations promote the creation of multifunctional signaling complexes that activate various intracellular signaling pathways, including PI3K-Akt and Mitogen Activated Protein kinase (MAPK) [90]. The most important path for glucose metabolic regulation is the PI3K-Akt signal for promoting Glucose Transporter 4 (GLUT4) translocalization to the cell membrane, increasing glucose infiltration into the cell. In the MAPK pathway, on the other hand, it is evident that the path contributes to cell growth and survival by ensuring there is a regulated gene expression to govern the rates of cell proliferation as well as the rate of apoptosis. As such, critical functioning in cells, the MAPK pathways provide evidence of intricate specifics and tight guilds of operations in pathways with consideration to insulin receptor signaling.

### 4.9 New Insights and Perspectives

The discovery of insulin a century ago and its application in treating human disease marked a watershed moment in medicine, one that not only revolutionized clinical practice but also deepened our understanding of metabolic regulation at the molecular level [91]. This breakthrough paved the way for the identification of the insulin receptor (IR) and its downstream signaling cascade, clarifying key regulatory processes and pathogenic mechanisms involved in metabolic disorders. While extensive research has elucidated much about this pathway, recent studies have unveiled new regulatory mechanisms, promising therapeutic targets, and emerging factors that influence insulin signaling, thereby offering innovative avenues to combat insulin resistance and type 2 diabetes [91, 92].

One pivotal regulatory mechanism involves tyrosine phosphatases, particularly PTP-1B and TCPTP, which negatively regulate insulin signaling by dephosphorylating the IR and insulin receptor substrate (IRS) proteins. Robust evidence from both experimental and clinical studies indicates that these phosphatases are markedly upregulated in conditions of chronic hyperinsulinemia and obesity, states frequently linked to excessive caloric intake, thereby contributing to insulin resistance, even within the hypothalamus [93]. Moreover, selective inhibition of PTP-1B has shown promising results in preclinical models and early clinical trials, suggesting that targeted therapies could restore insulin sensitivity and mitigate the metabolic dysfunctions associated with diabetes and obesity [94].

In parallel, the role of magnesium (Mg^2+^) in insulin signaling has attracted growing interest. Numerous studies have demonstrated that intracellular Mg^2+^ is essential for the optimal activity of tyrosine kinases, which are critical for IR autophosphorylation and the subsequent activation of downstream signaling pathways [95]. Emerging evidence suggests that chronic Mg^2+^ deficiency not only impairs insulin action but may also contribute to β-cell dysfunction, accelerating the progression of diabetes. This connection highlights the therapeutic potential of Mg^2+^ supplementation as a novel strategy for managing insulin resistance and preserving pancreatic function [96]. Insulin resistance is associated with oxidative stress, mitochondrial dysfunction, and inflammatory status. In addition to magnesium, cerium oxide nanoparticles are being considered as promising nanomaterials with antioxidant and anti-inflammatory properties, which may have an insulin-sensitizing effect, specifically in adipose tissue and skeletal muscle, in relation to mitochondrial function [97]. A recent review on these materials has been published, reporting the antioxidant action and clinical benefits using cerium oxide nanoparticles [98].

Beyond the classical regulation by phosphorylation, recent findings emphasize the importance of additional post-translational modifications, such as ubiquitination and acetylation, in modulating insulin receptor activity [99-102]. These modifications can affect the receptor’s stability, cellular localization, and interactions with other signaling molecules, thereby providing new molecular targets for enhancing insulin sensitivity in metabolic disorders [103-105].

## 5 STUDY LIMITATIONS

Despite the systematic approach used in this review, some limitations persist. The literature search was limited to three databases (PubMed/Medline, Scopus, and Web of Science) and confined to articles published between 2019 and 2024, which may have excluded relevant studies outside these parameters. Furthermore, the strict inclusion criteria and manual screening could have introduced selection bias, given the heterogeneity of experimental designs and reporting standards.

In addition, while the review meticulously delineates the molecular interactions and phosphorylation events critical to insulin receptor activation, it is well known that the multifaceted nature of insulin signaling encompasses additional regulatory mechanisms, such as other post-translational modifications and receptors with alternate signaling pathways that were not exhaustively explored. Such omissions highlight the need for further research to integrate these dimensions and to clarify their contributory roles in both physiological and pathophysiological contexts.

Finally, the translation of molecular-level insights to clinical outcomes remains challenging, as *in vivo* systems present additional layers of complexity that are not fully recapitulated by *in vitro* models or isolated biochemical analyses. Future investigations employing integrative methodologies, encompassing computational modeling, advanced imaging, and robust *in vivo* experimentation, are warranted to validate and expand upon the mechanistic understanding provided herein.

## CONCLUSION

The present review emphasizes chemical interactions of the insulin receptor (IR) leading to its activation and, in turn, to the signaling mechanisms that mediate metabolic control. After insulin binds the IR, a signaling pathway is activated, starting with the autophosphorylation of the central tyrosine residues inside the kinase catalytic domain. The phosphorylation of the receptor mediates its full activation and promotes the recruitment of intracellular signaling proteins responsible for glucose uptake, glycogen synthesis, and lipid metabolism. These include several critical residues within the kinase domain of the IR. Phosphorylation is also specific to the tyrosine residue at position 1162, which acts as a docking site to which other adaptor proteins possessing an SH2 domain will associate to form the signaling complex through which the insulin signal will be continued. This can be one of the reasons why the molecular interactions in IR would be proposed to have an increased understanding of the insulin signaling pathways and their regulation. This also puts great emphasis on the importance of amino acid interactions in keeping the metabolic homeostasis intact and giving enlightenment regarding the pathophysiology of insulin resistance and related metabolic disorders. Indeed, it is such a subtle interaction; if disturbed, this could lead to an impaired insulin signal and metabolic dysregulation, indicating potential therapeutic targets. Ultimately, the basis for activation and the mechanism of action of the insulin receptor are molecular interactions. This review stresses the importance of such interactions in the metabolic regulation domain. Future studies that build on these findings can be developed to use this essential insight in the construction of approaches to the design of targeted therapies for insulin sensitivity in treating metabolic diseases and, primarily, for better metabolic health outcomes.

In summary, a deeper understanding of the intricate chemical interactions between insulin and its receptor has not only led to the development of more effective insulin analogs but has also opened innovative therapeutic strategies aimed at countering insulin resistance on a molecular level. By incorporating these emerging insights, future research holds the potential to revolutionize diabetes treatment and metabolic disease management, paving the way for more precise and efficacious interventions.

## Figures and Tables

**Fig. (1):**
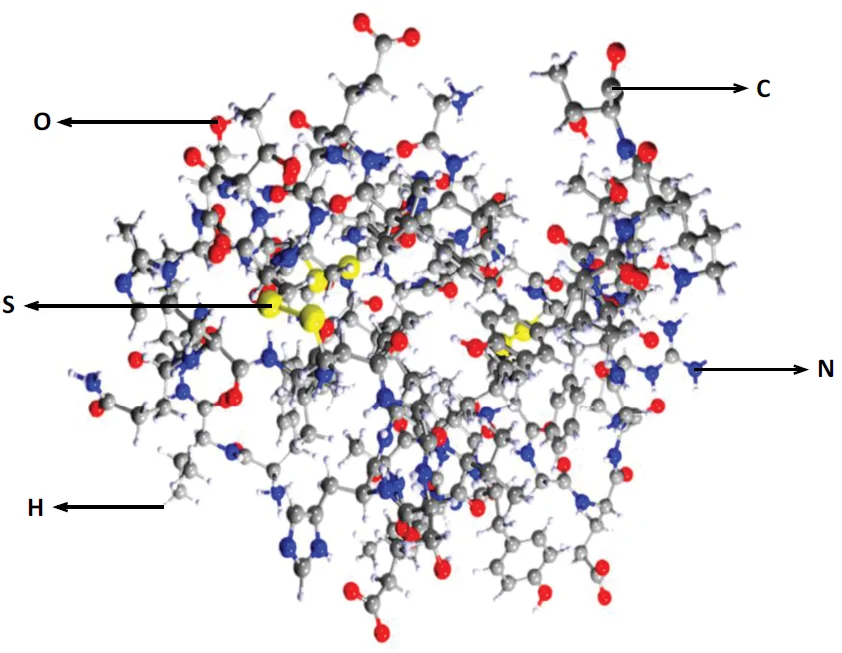
Molecular structure of the insulin peptide, showing the atomic spatial distribution of carbon, oxygen, hydrogen, nitrogen and sulfur.

**Fig. (2):**
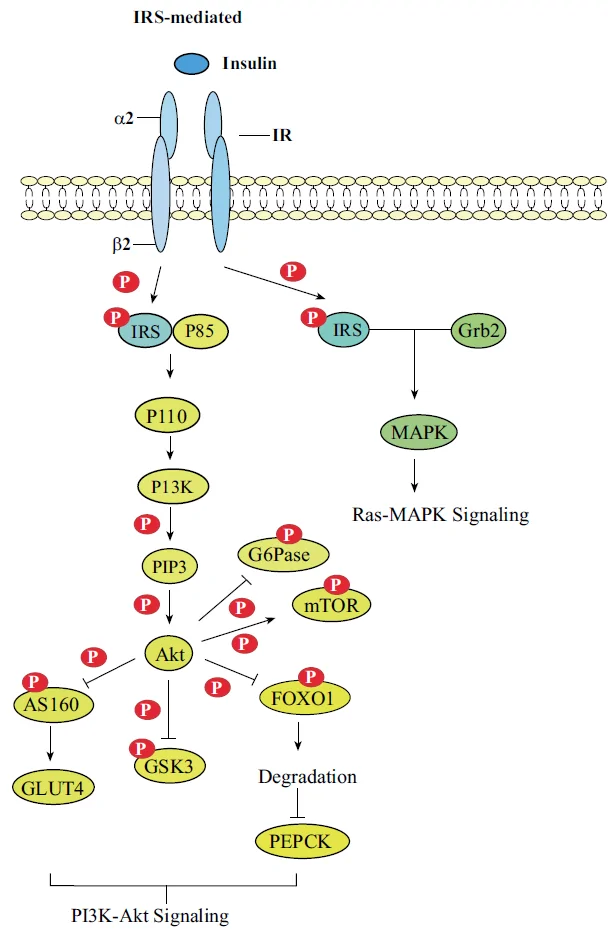
Simplified diagram of the insulin signaling pathway, illustrating key interactions and major components.

**Fig. (3):**
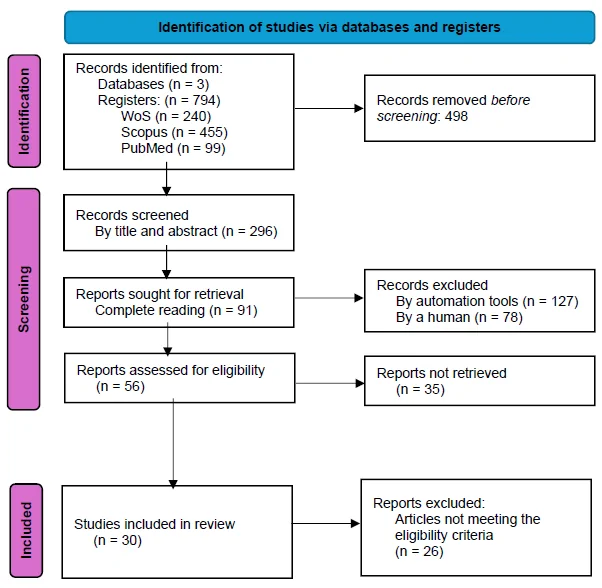
PRISMA Flow Diagram with the results of the article selection.

**Fig. (4):**
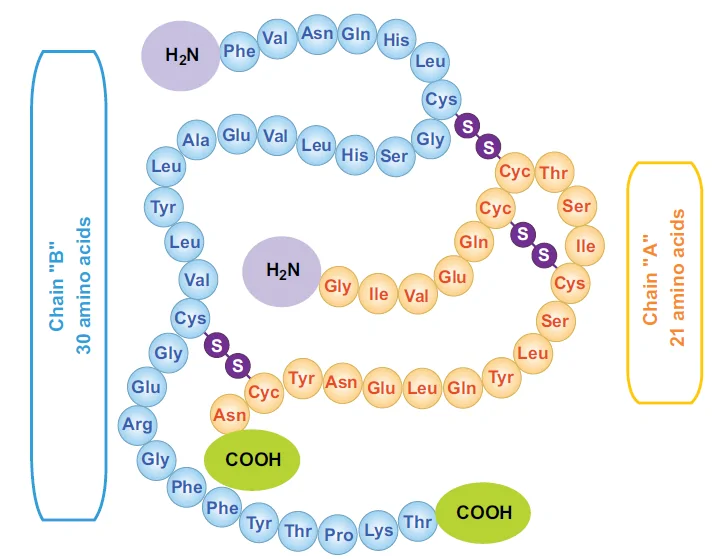
Insulin is composed of two polypeptide chains: A, which has 21 amino acid residues, and B, which has 30 amino acid residues. Chains A and B are connected by disulfide bridges.

**Fig. (5):**
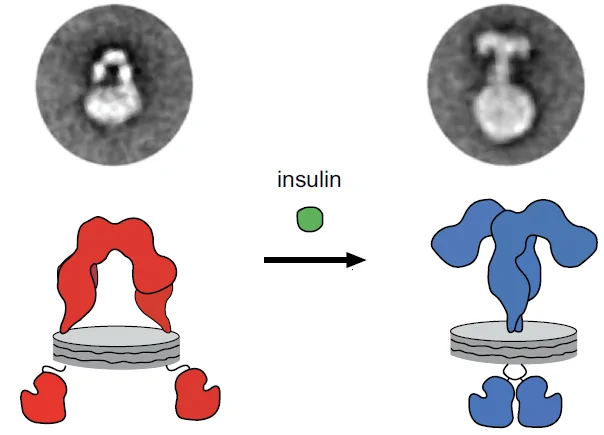
Insulin receptor in action, verified by single-particle Electron Microscopy (EM) to visualize a conformational change induced by insulin that leads to receptor activation (reconstituted in lipid nanodiscs). The results showed that, in the absence of insulin, the receptor ectodomain adopts an inverted U shape, changing to a T shape after insulin binding.

**Fig. (6):**
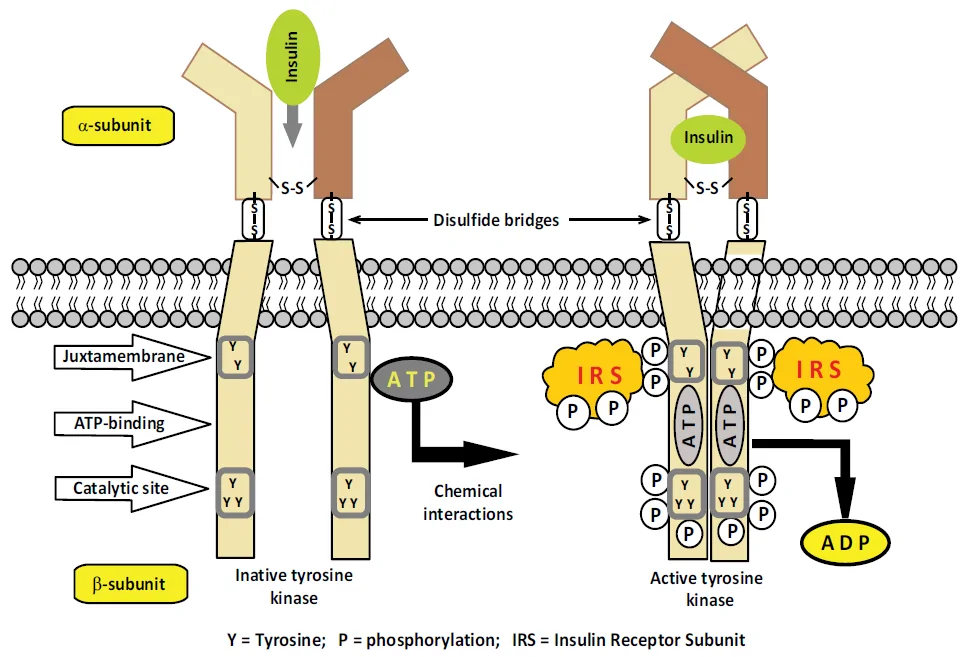
Activation of the insulin receptor through phosphorylation. On the left, the receptor is inactive, with unphosphorylated tyrosine (Y) residues. Insulin binding induces conformational changes, allowing interaction with ATP and subsequent phosphorylation of tyrosine residues in the intracellular kinase domain (right). This process activates the signaling cascade *via* IRS proteins, which are essential for glucose metabolism regulation.

## LIST OF ABBREVIATIONS

3D: Three-dimensional
AC: Affinity Chromatography
ADP: Adenosine Diphosphate
AKT/PKB: Protein Kinase B
Ala: Alanine
Arg: Arginine
Asn: Asparagine
Asp: Aspartic Acid
ATP: Adenosine Triphosphate
C-lobe: C-terminal lobe
Cys: Cysteine
ELK-1: ETS Like-1
EM: Electron Microscopy
FISH: Fluorescence In Situ Hybridization
FnIII: Fibronectin Type III
Gln: Glutamine
Glu: Glutamic Acid
Gly: Glycine
GLUT4: Glucose Transporter 4
GRB2: Growth Factor Receptor-Bound Protein 2
Ile: Isoleucine
IP: Immunoprecipitation
IR: Insulin Receptor
IRS: Insulin Receptor Substrate
Leu: Leucine
Lys: Lysine
MAPK: Mitogen-Activated Protein Kinase
Met: Methionine
mRNA: Messenger Ribonucleic Acid
N-lobe: N-terminal lobe
p arm: Petit arm
Phe: Phenylalanine
Pi: Inorganic Phosphate
PI3K: Phosphatidylinositol-3-Kinase
PIP2: Phosphatidylinositol 4,5-Bisphosphate
PIP3: Phosphatidylinositol 3,4,5-Trisphosphate
PKB: Protein Kinase B
Pro: Proline
RAS: Rat Sarcoma Virus Protein
RTK: Receptor Tyrosine Kinase
Ser: Serine
SH2: Src Homology 2
SOS: Son of Sevenless Protein
SRF: Serum Response Factor
Thr: Threonine
TK: Tyrosine Kinase
Trp: Tryptophan
Tyr: Tyrosine
Val: Valine

## STANDARDS OF REPORTING

PRISMA guidelines and methodology were followed.

## SUPPLEMENTARY MATERIAL

PRISMA checklist is available as supplementary material on the publisher’s website along with the published article.
