# Supplementary material for: Chemical Interactions Involved in Insulin Receptor Activation: Scoping Review
Source: Curr Diabetes Rev. 2025 Nov 3;22(6):E15733998373981. doi: 10.2174/0115733998373981250930135544 (PMC13714748; doi:10.2174/0115733998373981250930135544)
Supplement: Supplementary file 1 [file CDR-22-6-E15733998373981_SD1.pdf]

Supplementary Material

Chemical Interactions Involved in Insulin Receptor Activation: Scoping Review

Sofia Fernandes Coriolano Araujo[1,*], Stuart Handerson Rodrigues Costa[2] and Antonio Souza Araujo[3,*]

[1] *Faculty of Medical Science, University of Pernambuco, RecifePE, 50100-130, Brazil;* [2]*School of Chemistry and Molecular Bioscience, The University of Queensland, St. Lucia. QLD 4072, Australia;* [3]*Institute of Chemistry, Federal University of Rio Grande do Norte, NatalRN, 59078-970, Brazil*

PRISMA 2009 Checklist

Section/topic	#	Checklist item	Reported on page #
**TITLE**			
Title	1	Identify the report as a systematic review, meta-analysis, or both.<br>Chemical interactions involved in insulin receptor activation: scoping review	02
**ABSTRACT**			
Structured summary	2	Provide a structured summary including, as applicable: background; objectives; data sources; study eligibility criteria, participants, and interventions; study appraisal and synthesis methods; results; limitations; conclusions and implications of key findings; systematic review registration number.	03
**INTRODUCTION**			
Rationale	3	Describe the rationale for the review in the context of what is already known.	04-06
Objectives	4	Provide an explicit statement of questions being addressed with reference to participants, interventions, comparisons, outcomes, and study design (PICOS).	07
**METHODS**			
Protocol and registration	5	Indicate if a review protocol exists, if and where it can be accessed (e.g., Web address), and, if available, provide registration information including registration number.	08
Eligibility criteria	6	Specify study characteristics (e.g., PICOS, length of follow-up) and report characteristics (e.g., years considered, language, publication status) used as criteria for eligibility, giving rationale.	09
Information sources	7	Describe all information sources (e.g., databases with dates of coverage, contact with study authors to identify additional studies) in the search and date last searched.	09
Search	8	Present full electronic search strategy for at least one database, including any limits used, such that it could be repeated.	09
Study selection	9	State the process for selecting studies (i.e., screening, eligibility, included in systematic review, and, if applicable, included in the meta-analysis).	10

Data collection process	10	Describe method of data extraction from reports (e.g., piloted forms, independently, in duplicate) and any processes for obtaining and con-firming data from investigators.	10
Data items	11	List and define all variables for which data were sought (e.g., PICOS, funding sources) and any assumptions and simplifications made.	10
Risk of bias in in-dividual studies	12	Describe methods used for assessing risk of bias of individual studies (including specification of whether this was done at the study or out-come level), and how this information is to be used in any data synt-hesis.	10
Summary measu-res	13	State the principal summary measures (e.g., risk ratio, difference in means).	10
Synthesis of re-sults	14	Describe the methods of handling data and combining results of stu-dies, if done, including measures of consistency (e.g., I^2) for each me-ta-analysis.	10

Section/topic	#	Checklist item	Reported on page #
Risk of bias across studies	15	Specify any assessment of risk of bias that may affect the cumulative evidence (e.g., publication bias, selective reporting within studies).	10
Additional analy-ses	16	Describe methods of additional analyses (e.g., sensitivity or sub-group analyses, meta-regression), if done, indicating which were pre-specified.	10
**RESULTS**			
Study selection	17	Give numbers of studies screened, assessed for eligibility, and inclu-ded in the review, with reasons for exclusions at each stage, ideally with a flow diagram.	10
Study characteris-tics	18	For each study, present characteristics for which data were extracted (e.g., study size, PICOS, follow-up period) and provide the citations.	11
Risk of bias within studies	19	Present data on risk of bias of each study and, if available, any out-come level assessment (see item 12).	12
Results of indivi-dual studies	20	For all outcomes considered (benefits or harms), present, for each study: (a) simple summary data for each intervention group (b) effect estimates and confidence intervals, ideally with a forest plot.	12
Synthesis of re-sults	21	Present results of each meta-analysis done, including confidence intervals and measures of consistency.	13
Risk of bias across studies	22	Present results of any assessment of risk of bias across studies (see Item 15).	14-15
Additional analysis	23	Give results of additional analyses, if done (e.g., sensitivity or sub-group analyses, meta-regression [see Item 16]).	16-18
**DISCUSSION**			
Summary of evi-dence	24	Summarize the main findings including the strength of evidence for each main outcome; consider their relevance to key groups (e.g., healthcare providers, users, and policy makers).	19-31
Limitations	25	Discuss limitations at study and outcome level (e.g., risk of bias), and at review-level (e.g., incomplete retrieval of identified research, re-porting bias).	31-32
Conclusions	26	Provide a general interpretation of the results in the context of other evidence, and implications for future research.	32-33
**FUNDING**			

Funding	27	Describe sources of funding for the systematic review and other support (e.g., supply of data); role of funders for the systematic review.	34-35

From: Moher D, Liberati A, Tetzlaff J, Altman DG, The PRISMA Group (2009). Preferred Reporting Items for Systematic Reviews and Meta-Analyses: The PRISMA Statement. PLoS Med 6(7): e1000097. doi:10.1371/journal.pmed1000097

For more information, visit: **www.prisma-statement.org**.
